# Circular RNA hsa_circ_0003574 as a biomarker for prediction and diagnosis of ischemic stroke caused by intracranial atherosclerotic stenosis

**DOI:** 10.3389/fphar.2022.961866

**Published:** 2022-09-26

**Authors:** Lingfei Li, Xiaoli Si, Jie Ruan, Zhumei Ni, Xiaoqin Li, Hongfei Sang, Wenqing Xia, Jinyu Huang, Keqin Liu, Shan Lu, Lin Jiang, Anwen Shao, Congguo Yin

**Affiliations:** ^1^ Department of Neurology, Hangzhou First People's Hospital, Hangzhou, China; ^2^ The Department of Neurology, The Second Affiliated Hospital, School of Medicine, Zhejiang University, Hangzhou, China; ^3^ Department of Pharmacy, First Affiliated Hospital, College of Medicine, Zhejiang University, Hangzhou, China; ^4^ Fourth Clinical Medical College of Zhejiang Chinese Medical University, Hangzhou, China; ^5^ Department of Cardiology, Hangzhou First People’s Hospital, Hangzhou, China; ^6^ Department of Neuropsychology, Beijing Shijitan Hospital, Capital Medical University, Beijing, China; ^7^ Department of Neurosurgery, Second Affiliated Hospital, School of Medicine, Zhejiang University, Hangzhou, China; ^8^ Key Laboratory of Precise Treatment and Cinical Translational Research of Neurological Disease, Hangzhou, China

**Keywords:** intracranial atherosclerotic stenosis, circular RNA, biomarker, atherosclerotic stroke, circRNA-miRNA-mRNA network, therapeutic targets

## Abstract

**Background:** Intracranial atherosclerotic stenosis (ICAS) is a common cause of first and recurrent ischemic stroke worldwide. Circular RNAs (circRNA)s have been recently suggested as candidate biomarkers in diagnosing and prognosis of ischemic stroke. A few circRNAs even serve as therapeutic targets that improves neurological function after ischemic stroke. However, the roles of circRNAs in ICAS caused ischemic stroke (ICAS-stroke) have not been fully understood. Therefore, in this study, we attempted to find some clues by investigating the different expression profiles of circRNAs between patients diagnosed with ICAS-stroke and normal control (NC)s.

**Methods:** The OE Biotech Human ceRNA Microarray 4 × 180 K (47, 899 probes) screened circRNAs differentially expressed in peripheral blood in a discovery cohort (5 NCs versus five patients with ICAS-stroke). Afterwards, a validation cohort (31 NCs versus 48 patients with ICAS-stroke) was performed by quantitative polymerase chain reaction (qPCR). Gene Ontology (GO), Kyoto Encyclopedia of Genes and Genomes (KEGG) and CircRNA–microRNA-mRNA interaction network was performed to identify potential interactions with microRNAs and pathway-deregulated circRNAs.

**Results:** There were 244 circRNAs differentially expressed in patients diagnosed with ICAS-stroke compared with NCs [fold change (FC) ≥ 2.0 and *p*-value<0.05]. Among the 244 circRNAs, 5 circRNAs (hsa_circ_0003574, hsa_circ_0010509, hsa_circ_0026628, hsa_circ_0074057, hsa_circ_0016993) were selected for following verification by qPCR. Only hsa_circRNA_0003574 was significantly upregulated in patients than in NCs. GO analysis indicated that predicted target genes involved various biological processes, cellular components, and molecular functions. KEGG analysis showed that many genes were enriched within the arginine and proline metabolism, pyrimidine metabolism, arginine and proline metabolism, lysosome, cytokine-cytokine receptor interaction, and RNA transport. The circRNA-miRNA-mRNA network analysis show the miRNAs that has_circ_0003574 likely interacts with.

**Conclusion:** We observed that hsa_circRNA_0003574 is upregulated in patients with ICAS-stroke compared with NCs, indicating it may be a potential novel biomarker and therapeutic target for ICAS-stroke. In addition, we analyzed the laboratory results and found that homocysteine and glycosylated hemoglobin were elevated among ICAS-stroke patients. The relationship between hsa_circRNA_0003574 and these parameters requires further investigation.

## Introduction

Intracranial atherosclerotic stenosis (ICAS) is one of the most common causes of acute and recurrent ischemic stroke worldwide (Chimowitz et al., 2011), especially among the Chinese population (Wang et al., 2014). Before the onset of ischemic stroke, ICAS patients were always asymptomatic and diagnosed incidentally. Once ischemic stroke occurs, the neurological injury is devastating and hard to recover. Therefore, biomarkers depicting the existence of ICAS could help monitor vascular health and preventive therapy can be give before ischemic stroke happens. Recently, non-coding RNAs have been reported to be biomarkers for ICAS, including serum microRNA-137 (Xuan et al., 2021), plasma miR-126 and miR-143 (Gao et al., 2019). Moreover, in the animal model, the downregulation of lncRNA SNHG16 inhibits vascular smooth muscle cell proliferation and cerebral atherosclerosis migration, indicating the possibility developing new therapeutic methods. Besides serum biomarkers, magnetic resonance imaging facilitates imaging evidence as a biomarker. A recent study reported that the enhancement ratio and plaque steepness help predict recurrent ischemic cerebrovascular events for patients with ICAS (Yang et al., 2022).

Circular RNA (circRNA) is a novel type of non-coding RNA and is reported to be a biomarker for diagnosis and prognosis in ischemic stroke. In 2020, the Ostolaza team from Spain discovered that 219 circRNAs were differentially expressed within the blood of patients with aortic atherosclerosis compared with cardiogenic embolism (Ostolaza et al., 2020). A validation cohort of 50 patients established that circRNA_102,488 was significantly downregulated in the blood of the patients diagnosed with atherosclerotic ischemic stroke (*n* = 25) than those with cardiogenic embolism (*n* = 25). Further bioinformatics analysis observed that circRNA_102,488 could affect several pathways associated with ischemic stroke etiology, such as the biogenesis of fatty acids or the degradation of lysine (Ostolaza et al., 2020). In the same year, Zhang et al. observed that the expression of circ_0003204 in the extracellular vesicle of ICAS patients was significantly elevated, with a positive correlation between the expression level of circ_0003204 and cerebral atherosclerosis (Zhang et al., 2020). Following functional study confirm that circ_0003204 sponges miR-370, thus inhibiting the proliferation, migration, and capillary-like formation of human aortic endothelial cells exposed to oxidized low-density lipoprotein. Therefore, blockage of circ_0003204 is a potential therapeutic target for alleviating the abnormal phenotype of endothelial cells in atherosclerosis pathology (Zhang et al., 2020). Later in 2021, Li et al. reported that circRNA_0001599 expression was significantly upregulated in patients through atherosclerotic stroke and was positively correlated with the degree of neurological impairment and infarct size among the patients (Li et al., 2021). We hypothesize that circRNAs could be differentially expressed in patients with ICAS-stroke. Our study aimed to identify and validate the differentially expressed circRNAs as potential biomarkers to detect ICAS-stroke patients.

## Materials and methods

### Inclusion and exclusion criteria

The ethics committee of the Affiliated Hangzhou First People’s Hospital, Zhejiang University School of Medicine, approved the current research protocol (approval ID: 2020-037-01). The participants or their legally authorized representatives gave informed written consent to enroll in the study. Consecutive men and non-pregnant women diagnosed with ICAS-stroke were admitted immediately before treatment. Patients presented at our hospital with ischemic stroke caused by severe stenosis of intracranial arteries (70–90%) on computed tomography angiography (CTA), digital subtraction angiography (DAS), or magnetic resonance angiography (MRA) were recruited. Normal controls (NCs) were recruited from the ward, whose CTA, DSA, and MRA depicted no history of significant artery stenosis. Patients with extracranial atherosclerosis, active malignant diseases, and syphilis were excluded from the study. After inclusion, laboratory and radiological data were also collected.

### RNA isolation from peripheral venous blood

The total RNA from venous blood samples was isolated through a Norgen RNA isolation kit (Norgen, Canada) based on the manufacturer’s instructions and purified using an RNeasy Mini Kit (Qiagen, Germany). The integration of total RNA was determined using an Agilent Bioanalyzer 2100 (Agilent Technologies, Santa Clara, CA, US).

### Microarray expression of circRNAs

RNA samples of each group were used to generate biotinylated complementary RNA (cRNA) targets for the LC Human ceRNA array (4*180K, Design ID: 085,202). Moreover, the biotinylated cRNA targets were hybridized with the slides. After hybridization, slides were scanned through the Agilent Microarray Scanner G5761A (Agilent Technologies, Santa Clara, CA, US). Data were extracted using the Feature Extraction software 12.0.3.1 (Agilent Technologies, Santa Clara, CA, US), and the raw data were normalized with the Quantile algorithm. The microarray experiments were performed using the Agilent technologies protocol at LC Sciences Corporation (Hangzhou, China).

### Selection of circRNAs for qPCR validation

Among the 244 differentially expressed circRNAs screened by LC Human ceRNA array, 5 circRNAs (3 upregulated and 2 downregulated) were selected for qPCR validation. The selection criteria fundamentally based on the principle of smaller *p*-value (<0.05) and larger FC. Moreover, there parameters are also considered: 1) exotic genomic location; 2) the length of circRNA length ranges from 400 bp to 1400 bp; 3) host gene function are related to pathophysiological process atherosclerosis, like endothelial function.

### Validation of array results by qPCR

Total RNA was isolated from the blood through the Norgen RNA isolation kit (Norgen, Canada). The complementary DNA (cDNA) was reverse transcribed from the 500 ng total RNA per sample using Superscript^®^ III First-Strand Synthesis Reverse Transcriptase (Invitrogen, Carlsbad, CA, United States) and primed using random primers. Real-time-qPCR (RT-qPCR) reactions were undergone in triplicate for each sample through the Power SYBR ^®^ Green PCR Master Mix (Invitrogen, Carlsbad, CA, United States) within a QuantStudio 12 K Flex Real-Time PCR System (Applied Biosystems, Foster City, CA, United States). The primers are listed in Table 3. The thermal cycling conditions include an initial denaturation step at 95°C for 3°min, followed by 39 cycles of 10 s at 95°C and 30 s at 58°C. The expression levels of circRNAs were normalized with the linear transcript, GAPDH, or convergent amplicon of the host gene.

### Gene Ontology (GO) and Kyoto Encyclopedia of Genes and Genomes (KEGG) pathway enrichment analyses

The Gene Ontology project provides a controlled vocabulary to describe gene and gene product attributes in any organism (http://www.geneontology.org). GO analysis cover three areas, cellular components, molecular functions, and biological processes, each category explains the biological function of genes at different levels. The KEGG pathway enrichment analysis is used for the degree of enrichment of differential genes in pathway terms.

### CircRNA-microRNA-mRNA interaction network

To find potential target of microRNAs, the target/microRNAs is predicted with home-made miRNA target prediction software based on TargetScan (http://www.targetscan.org/) and miRanda (http://www.microrna.org/microrna/home.do). Through merging the common targeted miRNAs, ceRNA network was constructed. There are three conditions must exist for ceRNA network to occur (Salmena et al., 2011). First, the relative concentration of the ceRNAs and their microRNAs is clearly important; second, the effectiveness of a ceRNA would depend on the number of microRNAs that it can “sponge”; third, not all the microRNA response elements on ceRNAs are equal. So, we only accept these ceRNA-pair relations passing some measures filtering. The bioinformatic analysis by Aksomics Inc. (Shanghai, China).

### Data analysis

All the experimental data were analyzed using SPSS software 24.0 (SPSS, United States). Differences in the expression levels between patients and NCs were assessed through a *t*-test or Mann-Whitney *U* test. The *p*-value < 0.05 was statistically significant.

## Results

### Microarray expression profile

**FIGURE 1:**
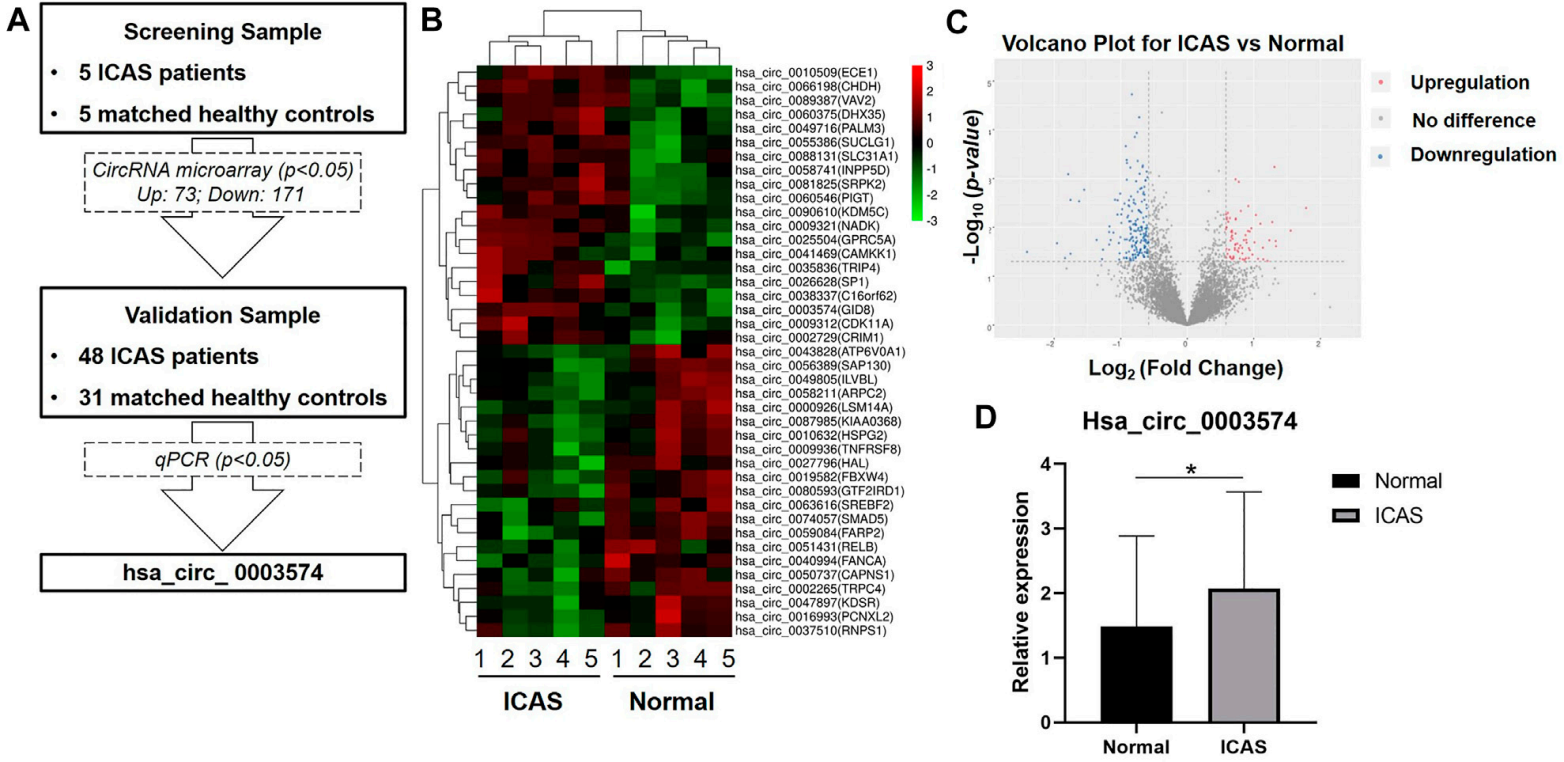
Hsa_circ_0003574 was upregulated in patients with ICAS-stroke compared with NC. **(A)** The flowchart illustrates the two-stage approach involving two independent cohorts for discovery and validation. **(B)** Compared with NCs, the top 20 upregulated and 21 downregulated circRNAs in ICAS-stroke patients. HeatMap and hierarchical clustering of the circRNA expression profile of five ICAS-stroke patients and NCs. **(C)** Volcano plot of differentially expressed, with 171 downregulated circRNAs (blue points) and 73 upregulated circRNAs (red points). **(D)** Expression of selected circRNAs among ICAS-stroke patients and NCs. Significant difference among the expression levels is denoted as *p < 0.05.

This study recruited five patients with ICAS-stroke and five age- and sex-matched NCs for the discovery cohort (Figure 1A). The characteristics are summarized in Table 1. The different expression patterns of circRNAs between ICAS-stroke patients and NCs were plotted through a hierarchical clustering heat map (Figure 1B) and a volcano plot (Figure 1C). There were 244 differentially expressed circRNAs between ICAS-stroke patients and the NCs (circRNAs with FC > 2 and *p* value<0.05). Among these, 73 circRNAs were upregulated, and 171 circRNAs were downregulated in ICAS-stroke patients than in controls.

**TABLE 1:** Demographic characteristics of ICAS-stroke patients and NCs among the discovery cohort.

Parameters	ICAS-stroke *n* = 5	NCs *n* = 5
Sex, male (no, %)	4 (80.0)	5 (100.0)
Age (years)	59.40 ± 8.08	59.80 ± 10.21
Smoking (no, %)	2 (40.0)	1 (20.0)
Hypertension (no, %)	5 (100.0)*	1 (20.0)
GLU (mmol/L)	6.46 ± 2.31	5.04 ± 0.55
SUA (mmol/L)	294.20 ± 64.25	339.20 ± 106.27
TC (mmol/L)	3.72 ± 0.65	4.09 ± 0.39
TG (mmol/L)	1.26 ± 0.57	0.97 ± 0.31
HDL (mmol/L)	1.10 ± 0.10	1.12 ± 0.29
LDL (mmol/L)	2.03 ± 0.51	2.40 ± 0.34
HCY (mmol/L)	12.84 ± 2.33	16.28 ± 3.48
GHB	6.46 ± 1.46	5.60 ± 0.45

ICAS-stroke, ischemic stroke caused by intracranial atherosclerotic stenosis; SD, standard deviation; no, number; GLU, blood glucose; SUA, serum uric acid; TC, total cholesterol; TG, triglyceride; HDL, high-density lipoprotein; LDL, low-density lipoprotein; HCY, homocysteine; GHB, Glycosylated hemoglobin. The data were represented as mean ± standard deviation. *p < 0.05

### Validation of differentially expressed circRNAs

We selected five circRNAs for qPCR validation of their expression based on the initial microarray analysis (Supplementary Table S1). The FC of 73 upregulated circRNAs ranges from 1.49 to 3.45 and we chose top 3 circRNAs with largest FC (hsa_circ_0003574, hsa_circ_0010509, hsa_circ_0026628). The lengths of the 3 circRNAs are within 400-1400bp and their functions are related to atherosclerotic process. The FC of 171 upregulated circRNAs ranges from 0.19 to 0.85, which is not large change. Therefore, we select hsa_circ_0074057 and hsa_circ_0016993 with the largest significant FC for validation. For validation, 48 ICAS-stroke patients (34 females and 14 males) and 31 age- and sex-matched NCs were recruited in this study. Demographic and clinical characteristics have been summarized in Table 2. Next, using a quantitative polymerase chain reaction, two downregulated circRNAs, and three upregulated circRNAs (FC > 1.2, *p* < 0.05) were validated in an independent validation cohort. The sequencings of primer are listed in Table 3, and the related information of the five genes is listed in Table 4. Among them, hsa_circ_0003574 (circular RNA GID8) depicted significantly elevated expression levels.

**TABLE 2:** Demographic characteristics of ICAS-stroke patients and NCs in the validation cohort.

Parameters	ICAS-stroke (*n* = 48)	NCs (*n* = 31)
Sex, male (no, %)	34 (69.4)	20 (60.6)
Age (years)	63.71 ± 11.90	61.45 ± 10.80
Smoking (no, %)	19 (38.8)	9 (27.3)
Hypertension (no, %)	41 (83.7)**	18 (54.5)
GLU (mmol/L)	5.83 ± 1.70**	4.89 ± 0.99
SUA (mmol/L)	293.29 ± 85.86	335.45 ± 117.12
TC (mmol/L)	3.85 ± 1.24	4.17 ± 1.10
TG (mmol/L)	1.54 ± 0.86	1.65 ± 1.23
HDL (mmol/L)	1.04 ± 0.24	1.14 ± 0.38
LDL (mmol/L)	2.18 ± 0.83	2.17 ± 0.82
HCY (mmol/L)	16.32 ± 9.54**	11.34 ± 2.42
GHB	6.06 ± 0.87***	5.52 ± 0.46

abrAbbreviationsICAS-stroke, ischemic stroke caused by intracranial atherosclerotic stenosis; SD, standard deviation; no, number; GLU, blood glucose; SUA, serum uric acid; TC, total cholesterol; TG, triglyceride; HDL, high-density lipoprotein; LDL, low-density lipoprotein; HCY, homocysteine; GHB, glycosylated hemoglobin. The data were represented as mean ± standard deviation. *p < 0.05, **p < 0.01

**TABLE 3:** List of primers for qPCR validation of the microarray results.

Target RNA	Primer sequence (5–3′)
hsa_circ_0003574	CCT​CAC​AGA​GAT​GGA​GCG​TA
hsa_circ_0003574	TTC​TCC​GCT​GCT​TCC​TTA​AA
hsa_circ_0010509	CCA​TCA​ACT​GGT​TGC​CTT​TT
hsa_circ_0010509	CTG​TCG​GTG​GTG​TTG​ATG​AG
hsa_circ_0026628	GGA​AGT​GGA​GGC​AAC​ATC​AT
hsa_circ_0026628	TGA​GAG​CTG​GGA​GTC​AAG​GT
hsa_circ_0074057	TGC​CAT​TTG​TGT​TTC​AGC​TC
hsa_circ_0074057	TCG​TGT​GGC​AGA​CAG​AAC​TC
hsa_circ_0016993	ATC​AGT​GGG​CCT​GAA​ACA​TC
hsa_circ_0016993	GGG​CCT​TCT​TTC​TGA​TTT​CC

**TABLE 4:** Selected circRNAs for qPCR validation, their microarray information, and the reason for validation.

CircRNA	*p* value (Benjamini Hochberg FDR)	Fold change	Regulation	Genomic location	Function	Reason for validation
hsa_circ_0003574	0.004	3.45	UP	*GID8*	A nuclear retention factor for β-catenin during wnt signaling (Lu et al., 2017)	Wnt/β-catenin pathway plays pivotal role in atherosclerosis (Vallee et al., 2019)
hsa_circ_0010509	0.012	2.93	UP	Enzyme that converts precursor big endothelin-1 to endothelin-1 (Takada et al., 1992)	Polymorphisms of *ECE1* is associated with stenosis of artery (Peltonen et al., 2009) and occurrence of ischemic stroke (Sui and He, 2014)
hsa_circ_0026628	0.025	2.52	UP	*SP1*	Transcription factor activates or represses transcription in response to physiological and pathological stimuli (Safe et al., 2014)	*SP1* pathway has been reported to be involved in atherosclerosis (Du et al., 2020)
hsa_circ_0074057	0.009	0.85	Down	*Smad5*	transforming growth factor beta signaling pathway that results in an inhibition of the proliferation of hematopoietic progenitor cells (Liu and Mao, 2004)	Mechanoresponsive Smad5 enhances miR-487a processing to promote vascular endothelial proliferation (Wang et al., 2021)
hsa_circ_0016993	0.026	0.85	Down	protein pecanex-like protein 2 Homo sapiens (*PCNXL2*)	may play a role in tumorigenesis with high microsatellite instability (Gudmundsson et al., 2017)	Not reported yet.

### Analysis of GO, KEGG and CircRNA-microRNA-mRNA interaction network

**FIGURE 2:**
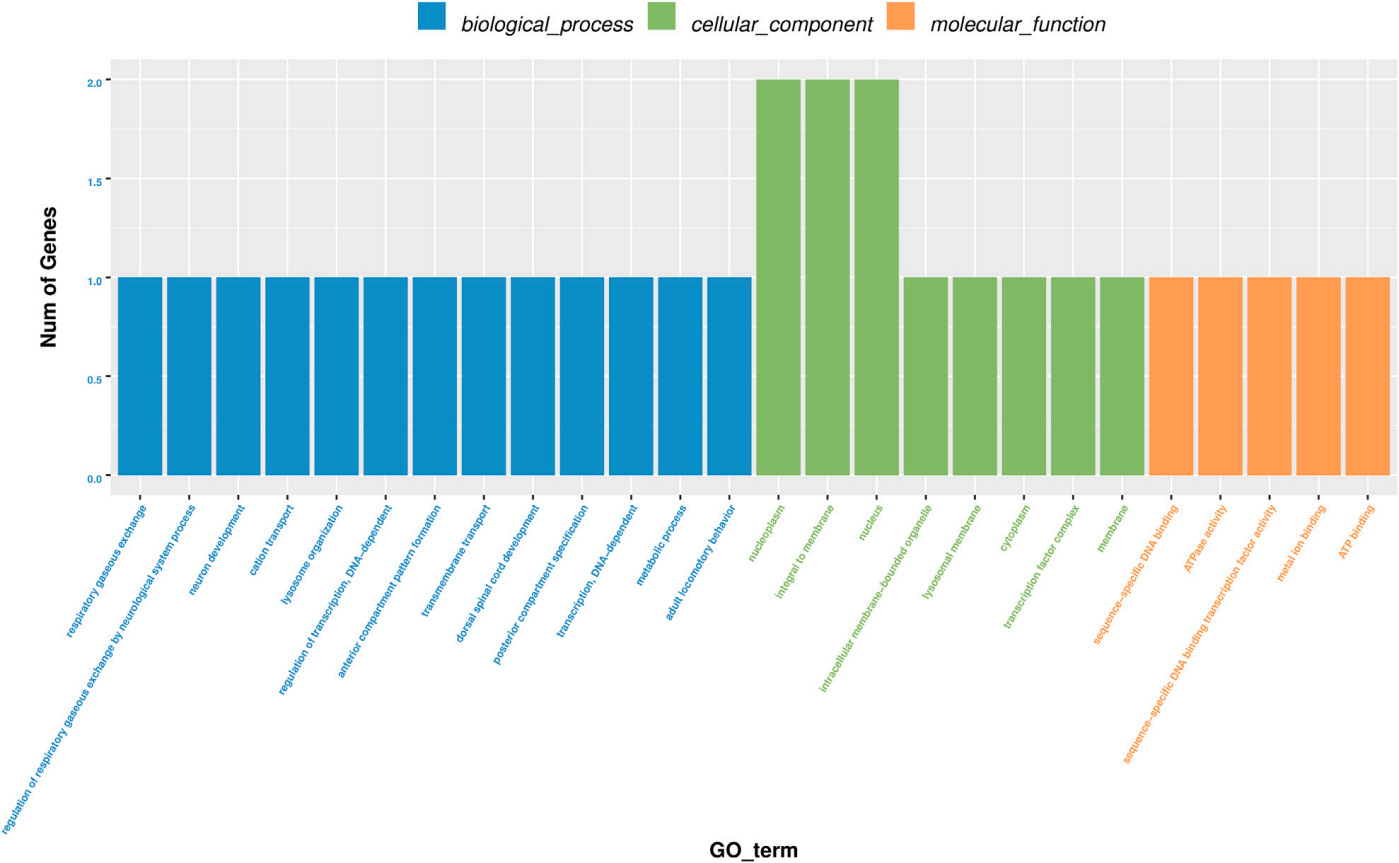
GO analysis. The histogram of GO enrichment analysis results affects the distribution of differential genes on GO terms enriched using cellular components and molecular function during the biological process.

**FIGURE 3:**
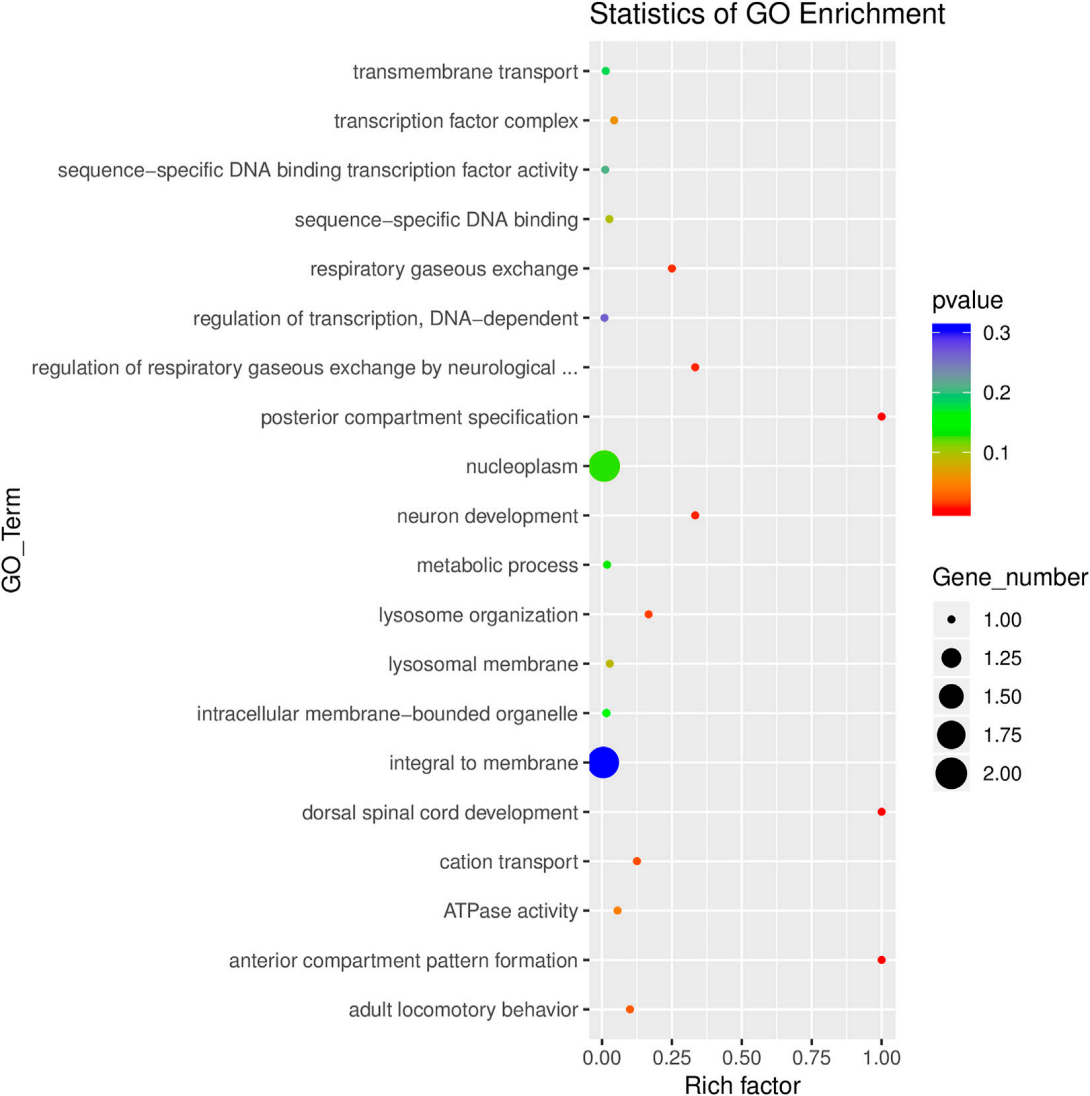
GO enrichment analysis results were presented through a scatter diagram. Rich factor depicts the number of differential genes located in the GO/the total number of genes in the GO. The more significant the Rich factor, the higher the GO enrichment degree.

**FIGURE 4:**
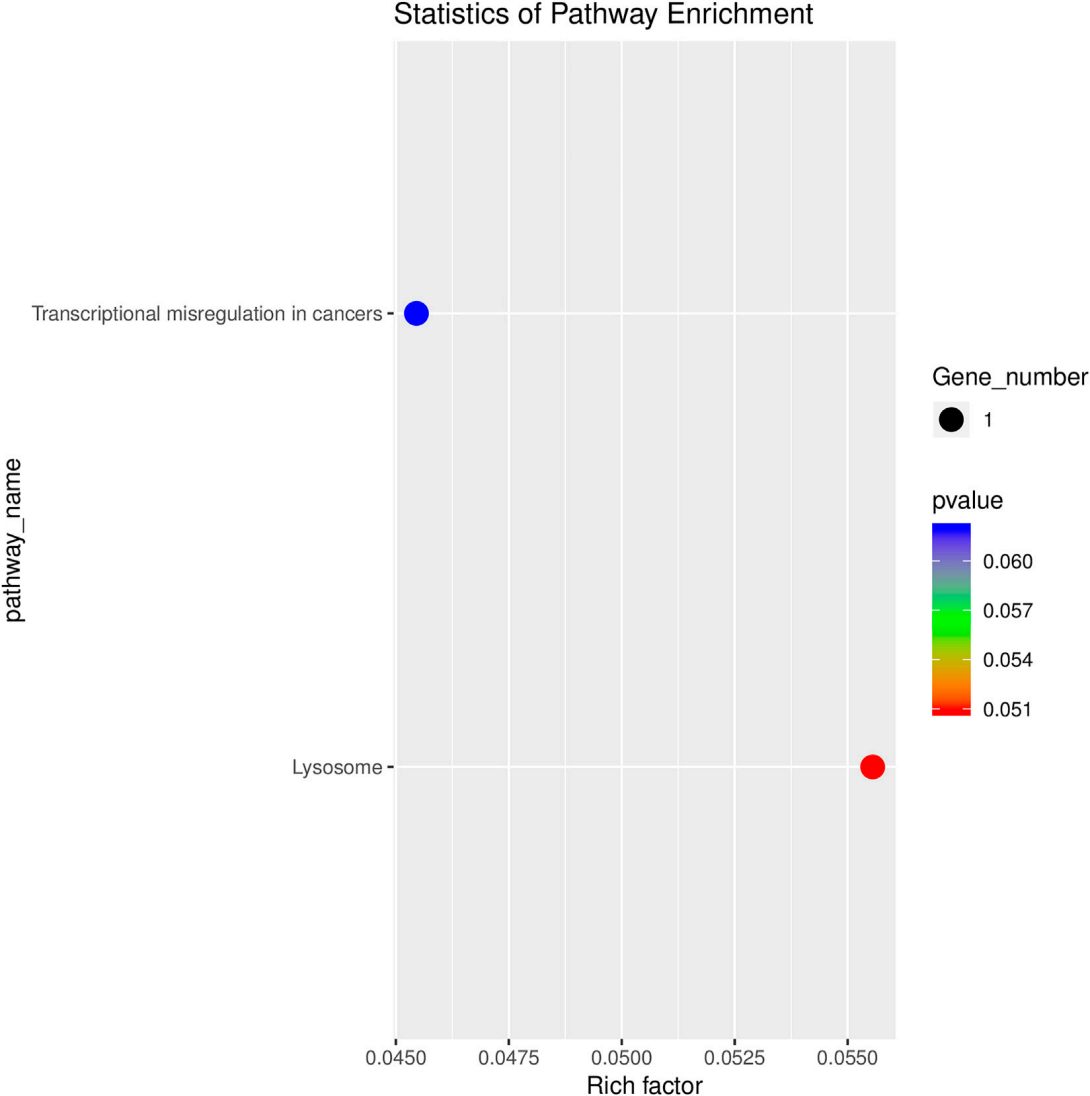
KEGG analysis. Bubble chart of the top 20 KEGG pathway analyses for target genes of the differentially expressed circRNAs.

**FIGURE 5:**
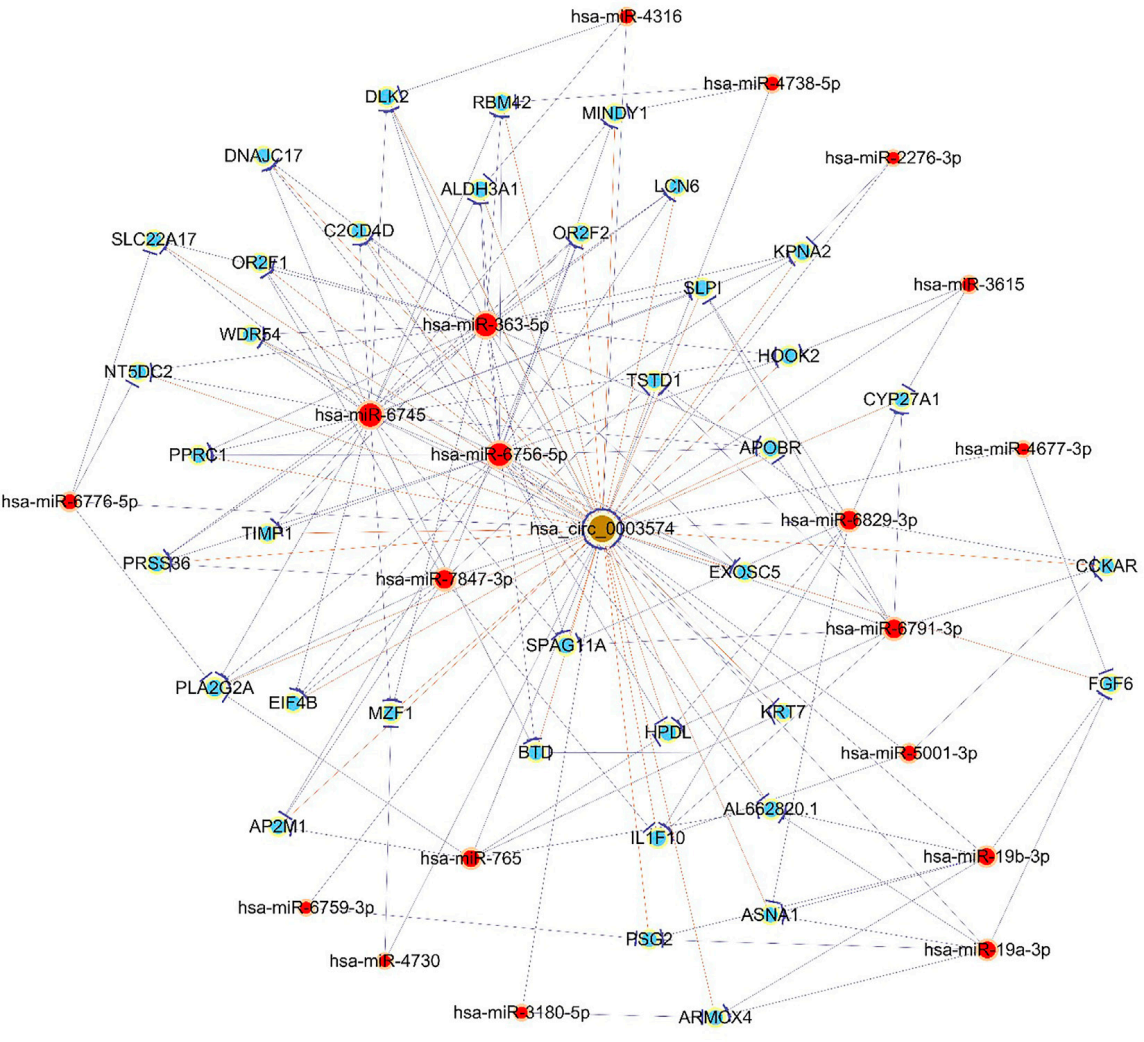
The constructed circRNA-miRNA-mRNA network. Hsa_circ_0003574 (brown nodes) was functionally associated with their targeted miRNAs in the network. Nodes with red color are microRNAs, and those with light-blue color are protein_coding RNAs. Edges with a T-shape arrow depict directed relationships. Edges without arrows depict undirected relationships (ceRNA relationship).

GO analyses revealed that these circRNAs were associated with 13 biological processes, 8 cellular components, and 5 molecular functions (Figure 2). The KEGG pathway analysis depicted these circRNAs associated with pathways, including arginine and proline metabolism, pyrimidine metabolism, lysosome, cytokine-cytokine receptor interaction, and RNA transport (Figures 3, 4). For circRNA-microRNA-mRNA interaction network, 145 mRNAs were predicted. There are 19 miRNAs and downstream 36 mRNAs most likely interacting with circRNA_0003574 (Figure 5).

## Discussion

This study observed that the hsa_circ_0003574 expression level enhanced in ICAS-stroke patients compared with sex- and age-matched NCs. Hsa_circ_0003574 is a biomarker predicting the occurrence of ICAS-stroke.

The parental gene of hsa_circ_0003574 is glucose-induced degradation of protein eight homologs (GID8). It is also known as two hybrid-associated protein no.1 with RanBPM (Twa1) (Umeda et al., 2003). GID8 is a highly conserved protein in evolution, but the biological function is unclear. It first interacted with RanBPM (Ran-binding protein M) (Umeda et al., 2003). A few early studies reported that yeast lack GID8 are more resistant to macrophage killing and display elevated host virulence among immunocompromised mice (Childers et al., 2016). High expression of GID8 was associated with a poor prognosis of colorectal and gastric cancers (Lu et al., 2017; Xiong et al., 2019). Moreover, the dysregulation of GID8 was correlated with delayed neuropsychomotor development and seizures (Correa et al., 2020). Furthermore, Lu et al. observed that GID8 was a nuclear retention factor for β-catenin promoting nuclear accumulation in response to Wnt signaling within colorectal cells (Lu et al., 2017). Evidence describes a protective role for Wnt signaling in cholesterol trafficking and its accumulation in multiple tissues, such as the arterial wall. Further understanding of the Wnt signaling pathway and its molecular regulation mechanism provides a novel idea. Based on this study, GID8 functions inside the nucleus, consistent with our GO analysis.

In this study, we first reported that the hsa_circ_0003574 expression level enhanced in ICAS patients, which could be utilized as a biomarker predicting its occurrence.

CircRNAs are reported as novel diagnostic and therapeutic targets in ischemic stroke. It involves different pathophysiological processes of ischemic stroke, including neuroinflammation (Ostolaza et al., 2020), apoptosis (Zhang et al., 2020), atherosclerosis (Gao et al., 2019), and neurogenesis (Li et al., 2021). Therefore, hypothetically, understanding the role of hsa_circ_0003574 during the pathophysiological process of ICAS-stroke can identify therapeutic targets in ischemic stroke. The advantages of research are: 1. Patient recruitment criteria were strictly controlled. Patients within two cohorts were matched in age and sex. 2. Possible downstream mechanism was analyzed with bioinformatics tools for molecular research. However, there were a few limitations in our study. First, more patients should be recruited for our research to verify the significance of diagnosing asymptomatic ICAS, especially in analyzing the distribution of stenotic lesions. Second, the association between vascular risk factors and the expression level of hsa_circ_0003574 requires further analysis. Third, an extended follow-up should verify the significance of hsa_circ_0003574 expression in predicting stroke recurrence in asymptomatic ICAS patients. Fourth, although other 239 circRNAs (not selected for qPCR validation) have lower possibility to be significantly different if they are validated by qPCR, we cannot totally make a conclusion they are not potential biomarkers. Further experiments needed to be done to validate the values of these circRNAs in ICAS-stroke patients. Last, no single index with excellent positive predictive value, sensitivity, and specificity can directly determine stroke recurrence in ICAS-stroke patients. This is because atherosclerotic stroke is complex and multifactorial, and imaging alone cannot be feasible. Therefore, it is more practical to build a multi-modal model that includes the nature of the plaque, plus other factors of the patient: age, lipid levels, and other markers, all combined to form a model.

## Data Availability

The authors acknowledge that the data presented in this study must be deposited and made publicly available in an acceptable repository, prior to publication. Frontiers cannot accept a manuscript that does not adhere to our open data policies.
