# Supplementary figures and images for: Circular RNA hsa_circ_0003574 as a biomarker for prediction and diagnosis of ischemic stroke caused by intracranial atherosclerotic stenosis
Source: Front Pharmacol. 2022 Sep 26;13:961866. doi: 10.3389/fphar.2022.961866 (PMC9549117; doi:10.3389/fphar.2022.961866)

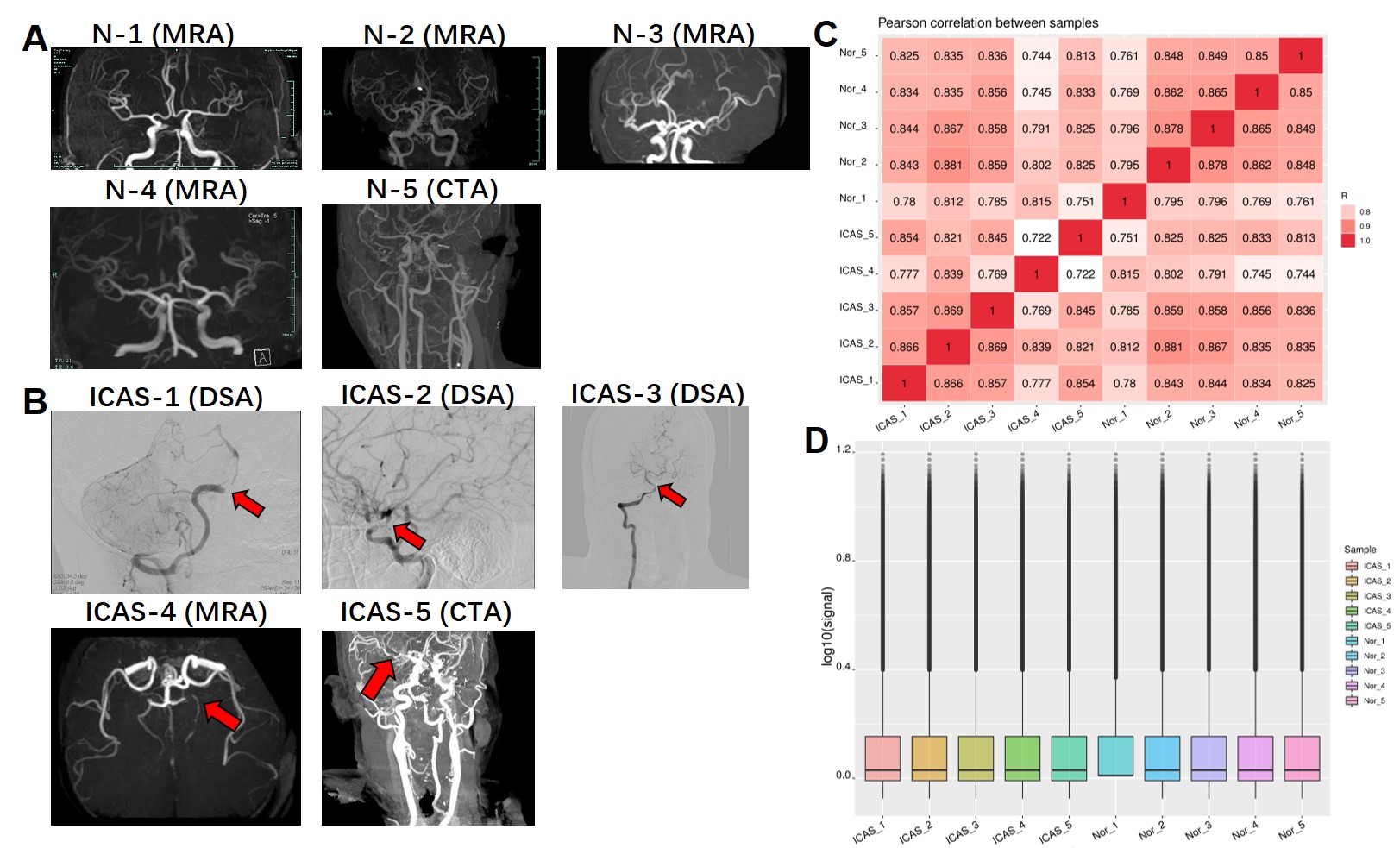

Supplement: Supplementary file 1 [file Image1.JPEG]

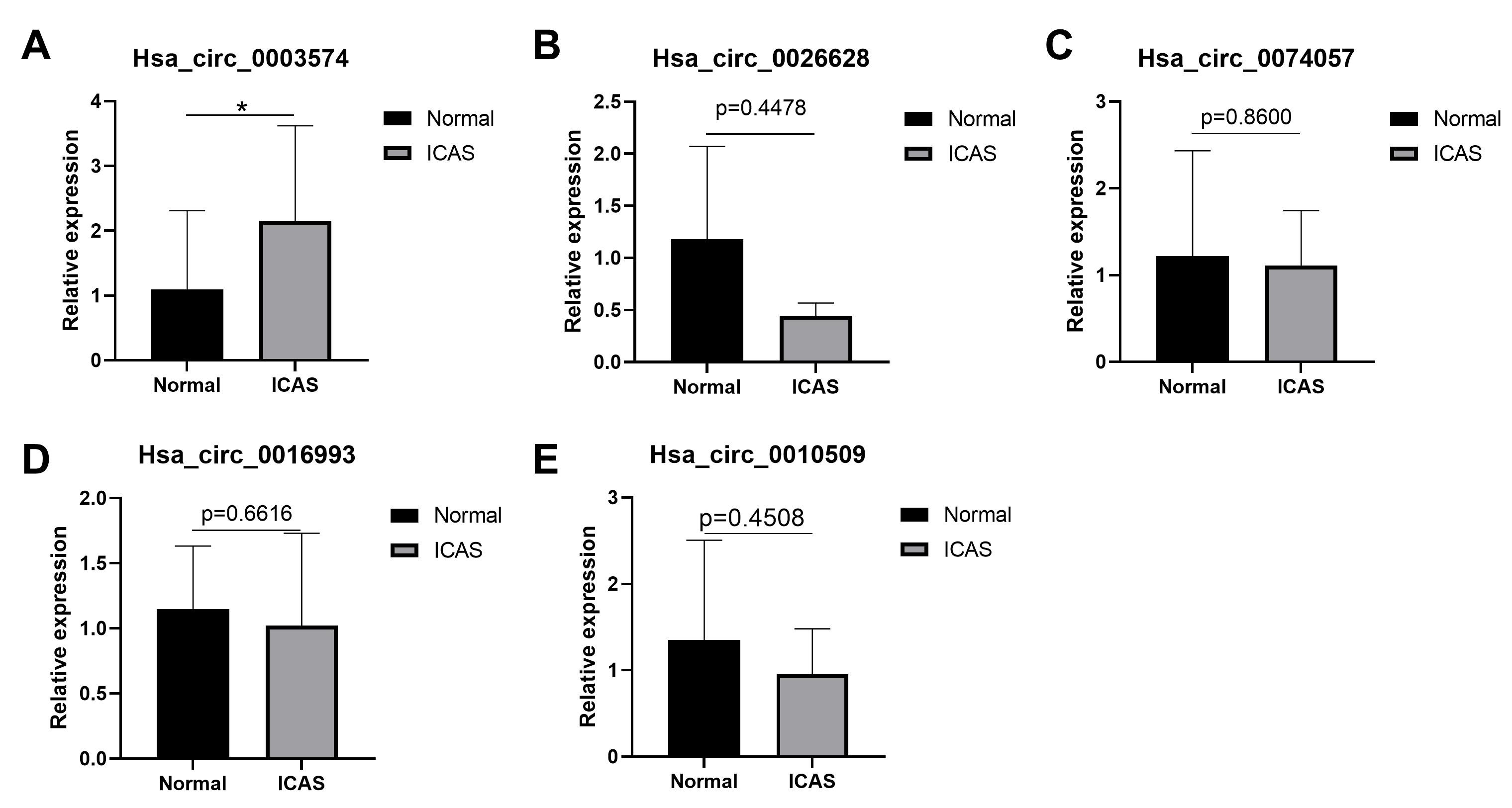

Supplement: Supplementary file 2 [file Image2.JPEG]
